# Supplementary material for: MAGinator enables accurate profiling of de novo MAGs with strain-level phylogenies
Source: Nat Commun. 2024 Jul 9;15:5734. doi: 10.1038/s41467-024-49958-8 (PMC11231285; doi:10.1038/s41467-024-49958-8)
Supplement: Supplementary file 1 — Supplementary Information [file 41467_2024_49958_MOESM1_ESM.pdf]

Supplementary Information: MAGinator enables accurate profiling of *de novo* MAGs with strain-level phylogenies

Table of contents

Supplementary Figure 1: MAGinator workflow..3
Supplementary Figure 2: Benchmark using OPAL...3
Supplementary Figure 3: Subspecies-resolved tracking of B. infantis metagenomes............. 4
Supplementary Figure 4: Subspecies-level analysis of B. longum in COPSAC2010.............. 5
Supplementary Figure 5: Stratification of StrainPhlAn clusters using relative abundance of MAGinator clusters - CHILD cohort..6
Supplementary Figure 6: Read mappings of B. infantis signature genes............................... 7
Supplementary Figure 7: SNV-level phylogenetic tree of a MAG cluster based on signature genes.. 8
Supplementary Figure 8: Synteny clusters and functional annotation of COPSAC2010.........9
Supplementary Table 1: Output generated by MAGinator.. 10
Supplementary Table 2: Parameters and default values of MAGinator.................................11
Supplementary Table 3: OPAL benchmark. Average completeness (%) across taxonomic ranks.. 13
Supplementary Table 4: OPAL benchmark. Average purity (%) across taxonomic ranks......13
Supplementary Table 5: Allele Frequency results for different metagenomics datasets........14

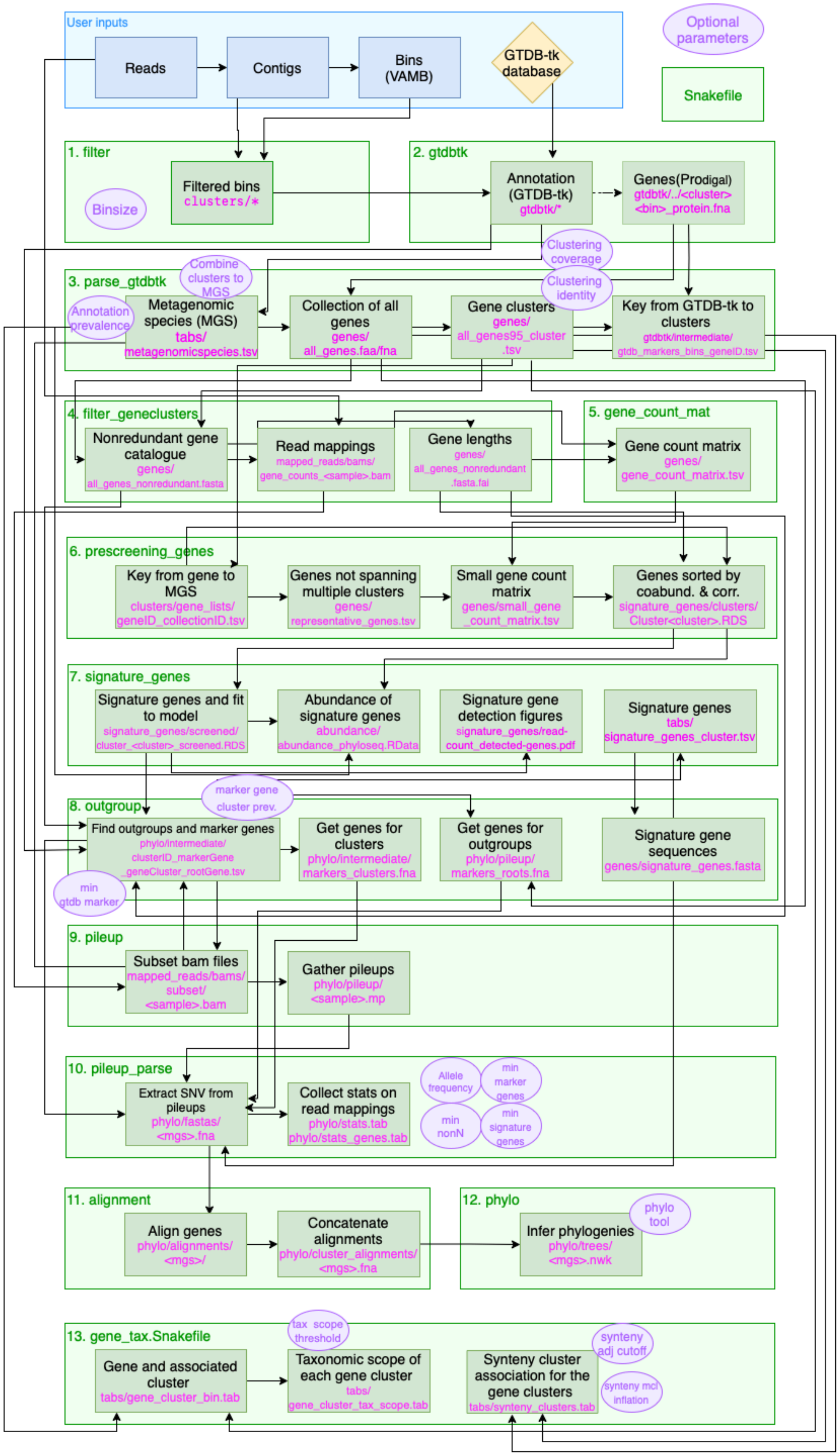

User inputs
Reads
Contigs
Bins (VAMB)
GTDB-tk database
Optional parameters
Snakefile
1. filter
Binsize
Filtered bins clusters/*
2. gtdbtk
Annotation (GTDB-tk) gtdbtk/*
Genes(Prodigal) gtdbtk/../<cluster><bin>_protein.fna
Clustering coverage
Clustering identity
3. parse_gtdbtk
Combine clusters to MGS
Annotation prevalence
Metagenomic species (MGS) tabs/ metagenomicspecies.tsv
Collection of all genes genes/ all_genes.faa/fna
Gene clusters genes/ all_genes95_cluster.tsv
Key from GTDB-tk to clusters gtdbtk/intermediate/ gtdb_markers_bins_geneID.tsv
4. filter_geneclusters
Nonredundant gene catalogue genes/ all_genes_nonredundant.fasta
Read mappings mapped_reads/bams/ gene_counts_<sample>.bam
Gene lengths genes/ all_genes_nonredundant.fasta.fai
5. gene_count_mat
Gene count matrix genes/ gene_count_matrix.tsv
6. prescreening_genes
Key from gene to MGS clusters/gene_lists/ geneID_collectionID.tsv
Genes not spanning multiple clusters genes/ representative_genes.tsv
Small gene count matrix genes/small_gene_count_matrix.tsv
Genes sorted by coabund. & corr. signature_genes/clusters/ Cluster<cluster>.RDS
7. signature_genes
Signature genes and fit to model signature_genes/screened/ cluster_<cluster>_screened.RDS
Abundance of signature genes abundance/ abundance_phyloseq.RData
Signature gene detection figures signature_genes/read-count_detected-genes.pdf
Signature genes tabs/ signature_genes_cluster.tsv
marker gene cluster prev.
8. outgroup
Find outgroups and marker genes phylo/intermediate/ clusterID_markerGene geneCluster_rootGene.tsv
min gtdb marker
Get genes for clusters phylo/intermediate/ markers_clusters.fna
Get genes for outgroups phylo/pileup/ markers_roots.fna
Signature gene sequences genes/signature_genes.fasta
9. pileup
Subset bam files mapped_reads/bams/ subset/ <sample>.bam
Gather pileups phylo/pileup/ <sample>.mp
10. pileup_parse
Extract SNV from pileups phylo/fastas/ <mgs>.fna
Collect stats on read mappings phylo/stats.tab phylo/stats_genes.tab
Allele frequency
min marker genes
min nonN
min signature genes
11. alignment
Align genes phylo/alignments/ <mgs>/
Concatenate alignments phylo/cluster_alignments/ <mgs>.fna
12. phylo
Infer phylogenies phylo/trees/ <mgs>.nwk
phylo tool
13. gene_tax.Snakefile
Gene and associated cluster tabs/gene_cluster_bin.tab
tax scope threshold
Taxonomic scope of each gene cluster tabs/ gene_cluster_tax_scope.tab
Synteny cluster association for the gene clusters tabs/synteny_clusters.tab
synteny adj cutoff
synteny mcl inflation

Supplementary Figure 1: *MAGinator workflow.*

A light green box indicates a snakefile and the darker green box indicates a deliverable (directory or file). The purple circles indicate user configurable parameters. The arrow indicates data dependencies, where the flow of information from one file is used to create the file it points towards.

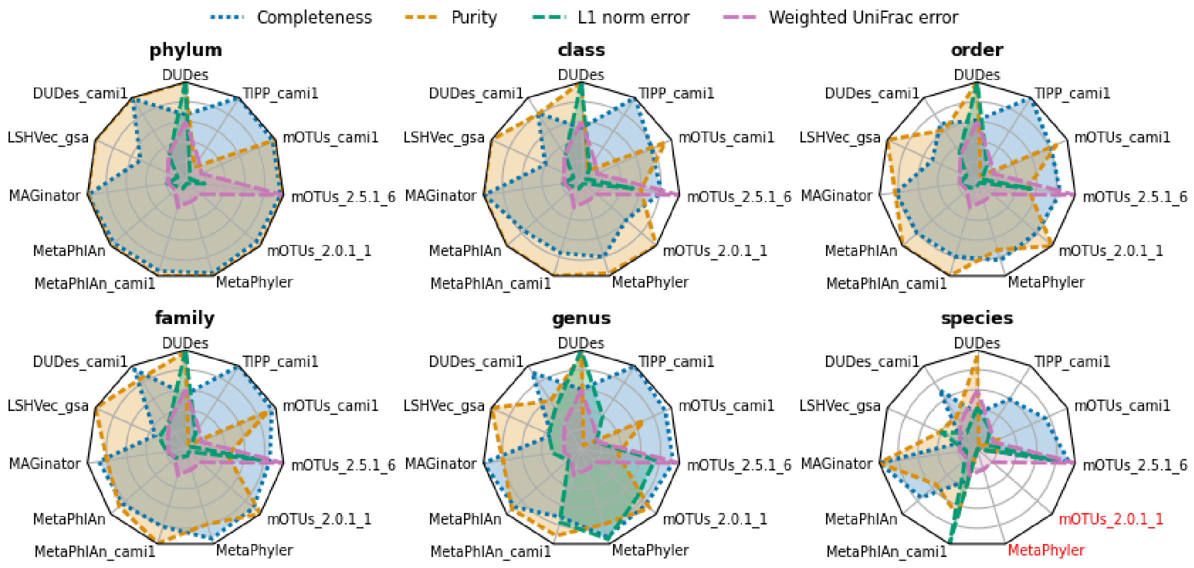

Supplementary Figure 2*: Benchmark using OPAL*

Comparing taxonomic profiling results for the CAMI strain-madness data set. Metrics for the relative abundance of the profiles are calculated across samples for Completeness, Purity, L1 norm error and Weighted UniFrac error and are shown in a Spider-plot for the taxonomic ranks between phylum and species-level. The tools indicated with red means no data was available for that rank. Source data are provided as a Source Data file.

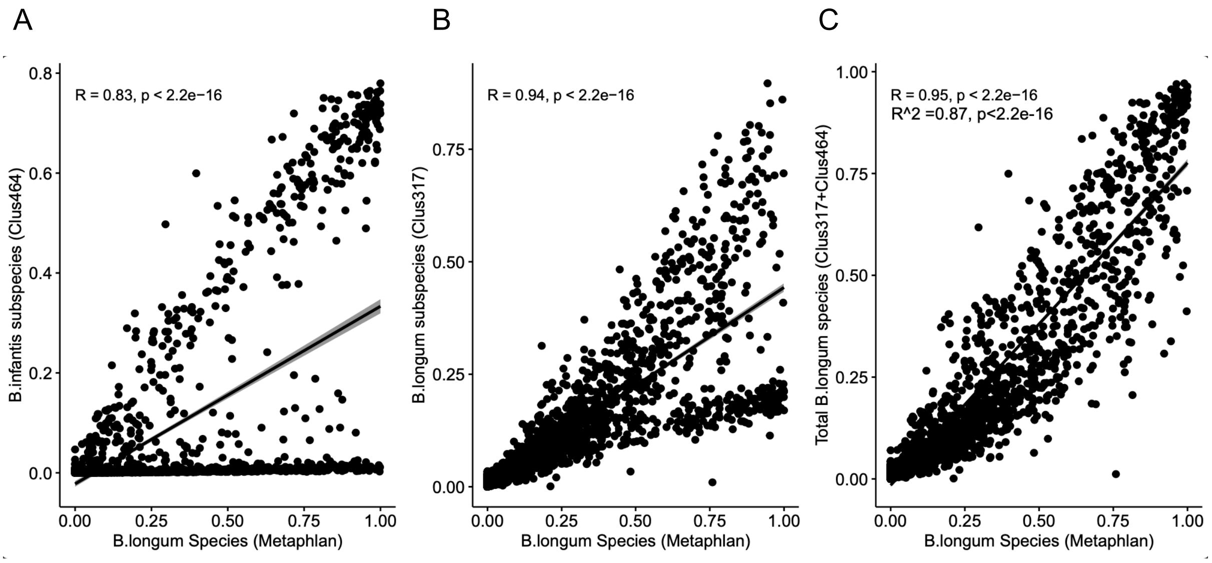

Supplementary Figure 3: *Subspecies-resolved tracking of B. infantis metagenomes*

The relationship between the relative abundance for the *B. longum* species in the CHILD dataset found with MetaPhlAn and MAGinator's representative clusters for (A) *B. longum* (B) *B. infantis* (C) Both abundances added together. Each dot indicates a sample. Source data are provided as a Source Data file.

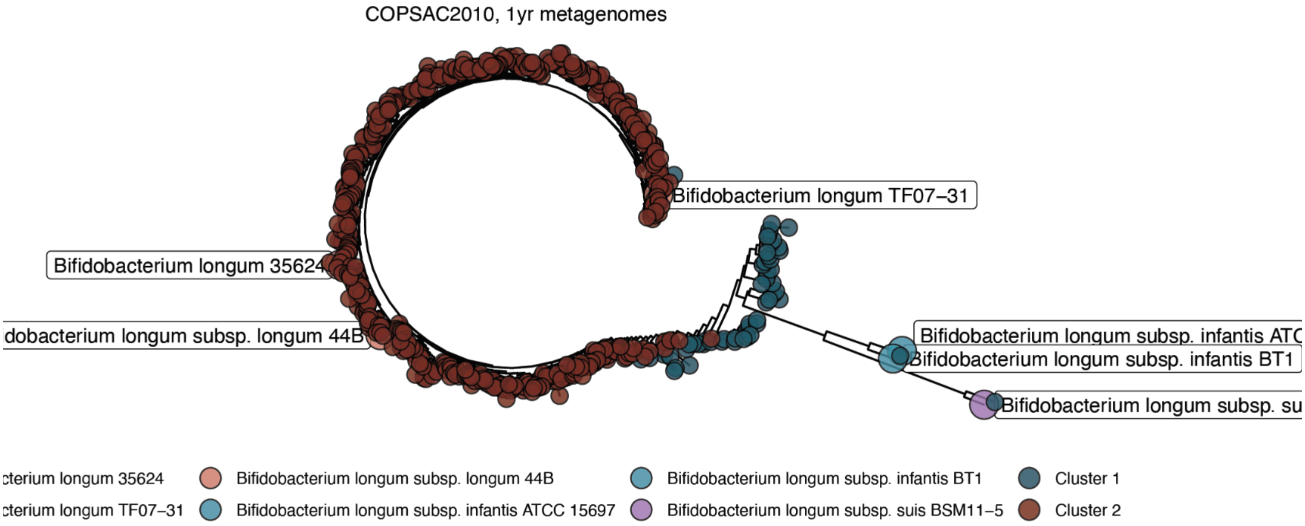

Supplementary Figure 4*: Subspecies-level analysis of B. longum* in COPSAC$_{2010}$

StrainPhlAn phylogenetic tree of samples based on SNVs of B. longum markers, resulting in 2 clades. Dots represent samples with sufficient marker coverage as well as the 6 references. Cluster 1 indicates *B. infantis* and Cluster 2 indicates *B. longum.* Source data are provided as a Source Data file.

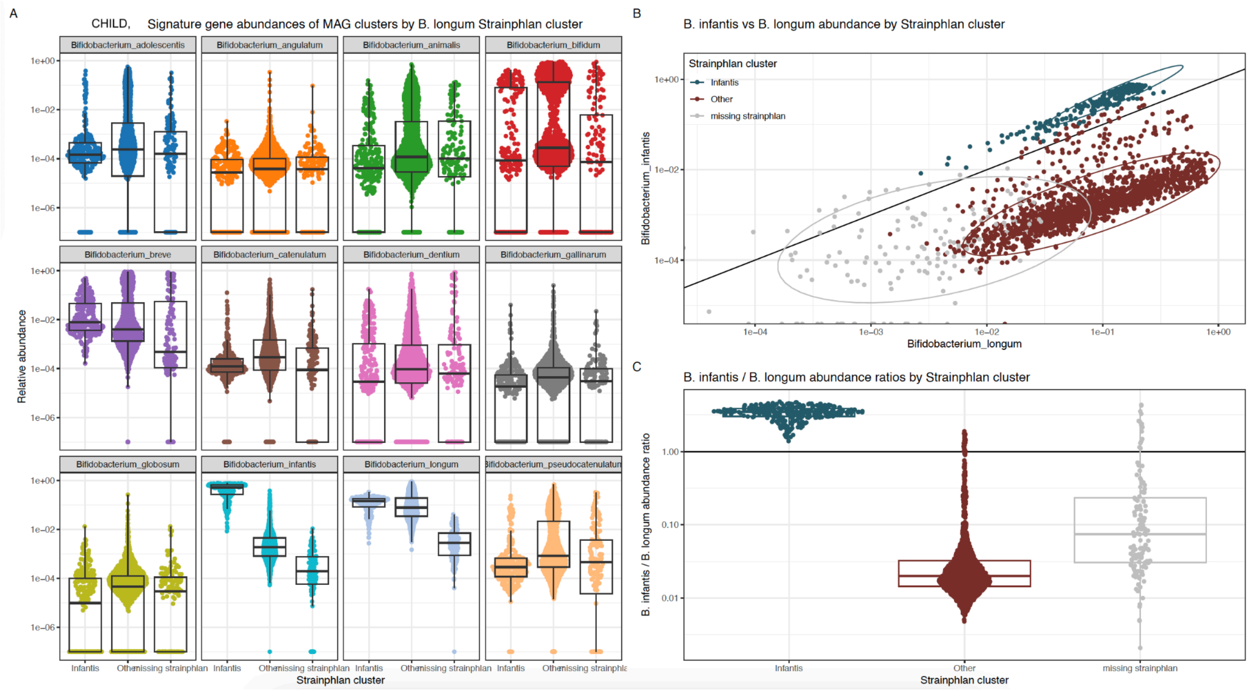

Supplementary Figure 5: *Stratification of StrainPhlAn clusters using relative abundance of MAGinator clusters - CHILD cohort*

Cluster 1 indicates B. infantis and Cluster 2 indicates B. longum.

(A) Relative abundance of StrainPhlAn clusters stratified by all Bifidobacterium clusters identified by MAGinator (B) Relative abundance of B. infantis and B. longum identified with MAGinator coloured by StrainPhlAn cluster. (C) The ratio of B. infantis to B. longum is displayed for the StrainPhlAn clusters. Source data are provided as a Source Data file.

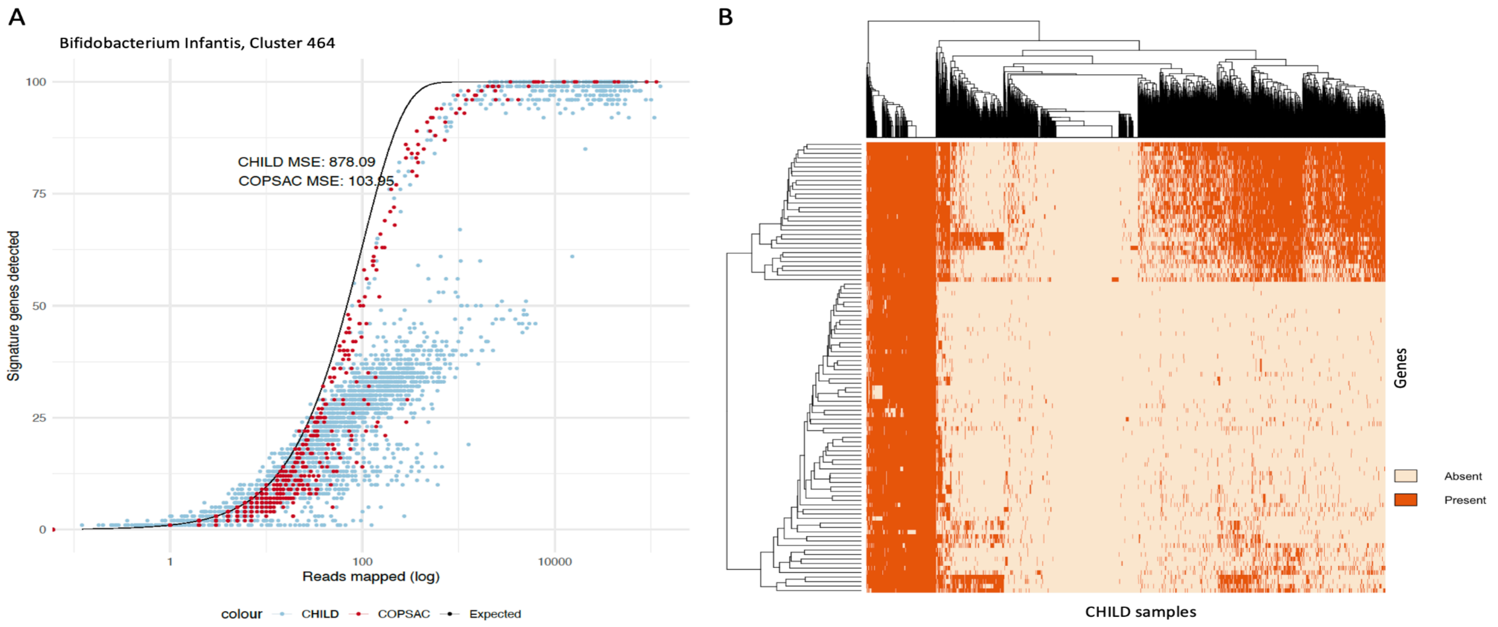

Supplementary Figure 6: *Read mappings of B. infantis signature genes*

(A) The number of reads mapped to the signature genes (defined in $COPSAC_{2010}$) of the *B. infantis* cluster is presented with the number of signature genes detected. Each dot is a sample. The red colour indicates $COPSAC_{2010}$ samples, the blue colour indicates CHILD samples. The black line indicates the expected distribution[1]. (B) Heatmap of the read mappings of the *B. infantis* signature genes for the CHILD samples. Source data are provided as a Source Data file.

[1] Zachariasen, T. *et al.* Identification of representative species-specific genes for abundance measurements. *Bioinforma. Adv.* **3**, vbad060 (2023).

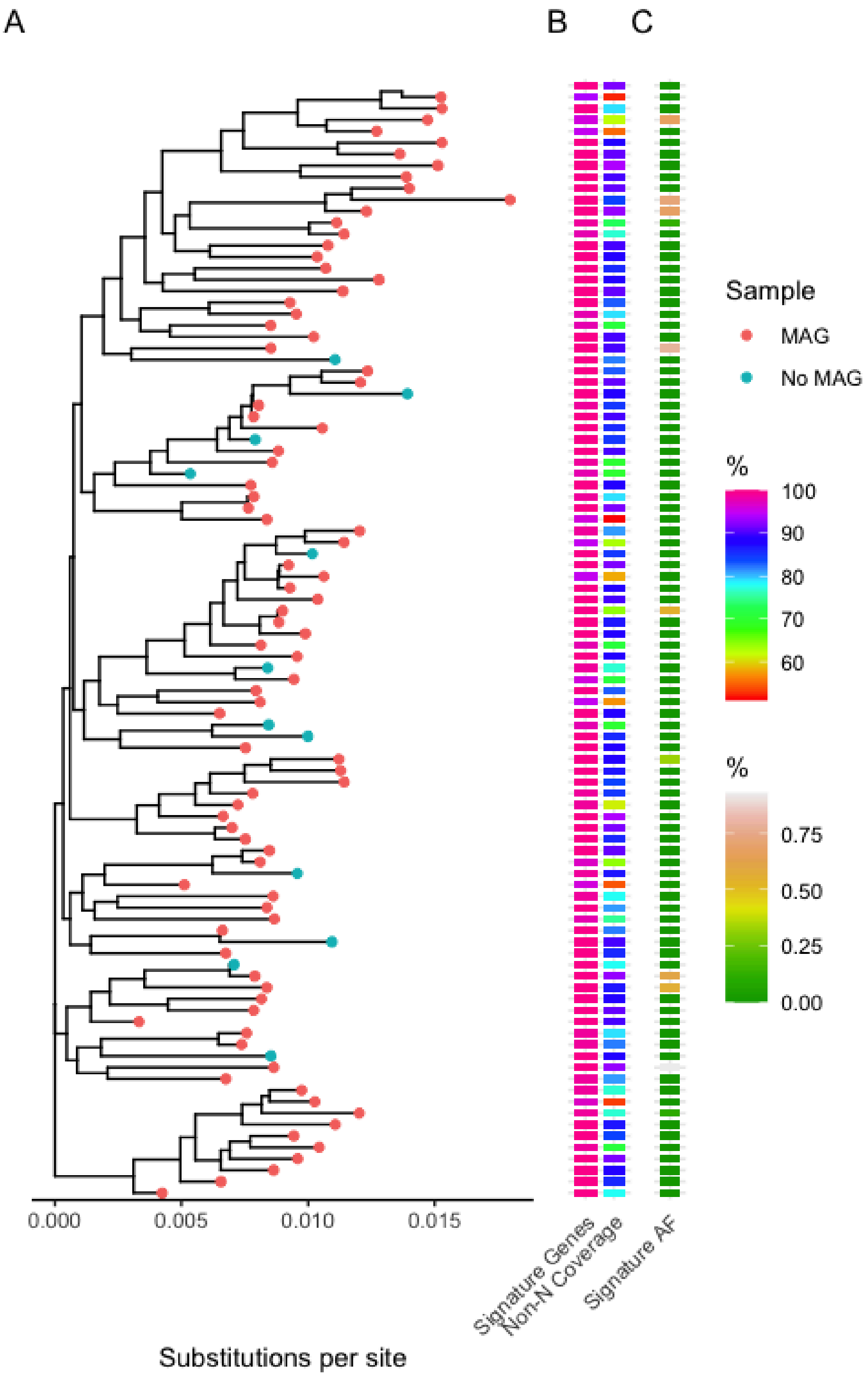

Supplementary Figure 7*: SNV-level phylogenetic tree of a MAG cluster based on signature genes*

A) Phylogenetic tree constructed from readmappings to the signature genes of a MAG cluster annotated as *Faecalibacterium* sp900758465 from COPSAC_{2010}. Tip colour indicates if the sample has a MAG. B) Heatmap showing how many of the 100 signature genes that are detected within the sample and the fraction of bases that are Non-N in the alignment of the reads to the signature gene sequence in each sample (%). C) Median frequency of bases in the signature gene alignment with mixed alleles (%). Only positions with read depth minimum 2 are included in the frequency. The numbers are normalised according to gene length. Source data are provided as a Source Data file.

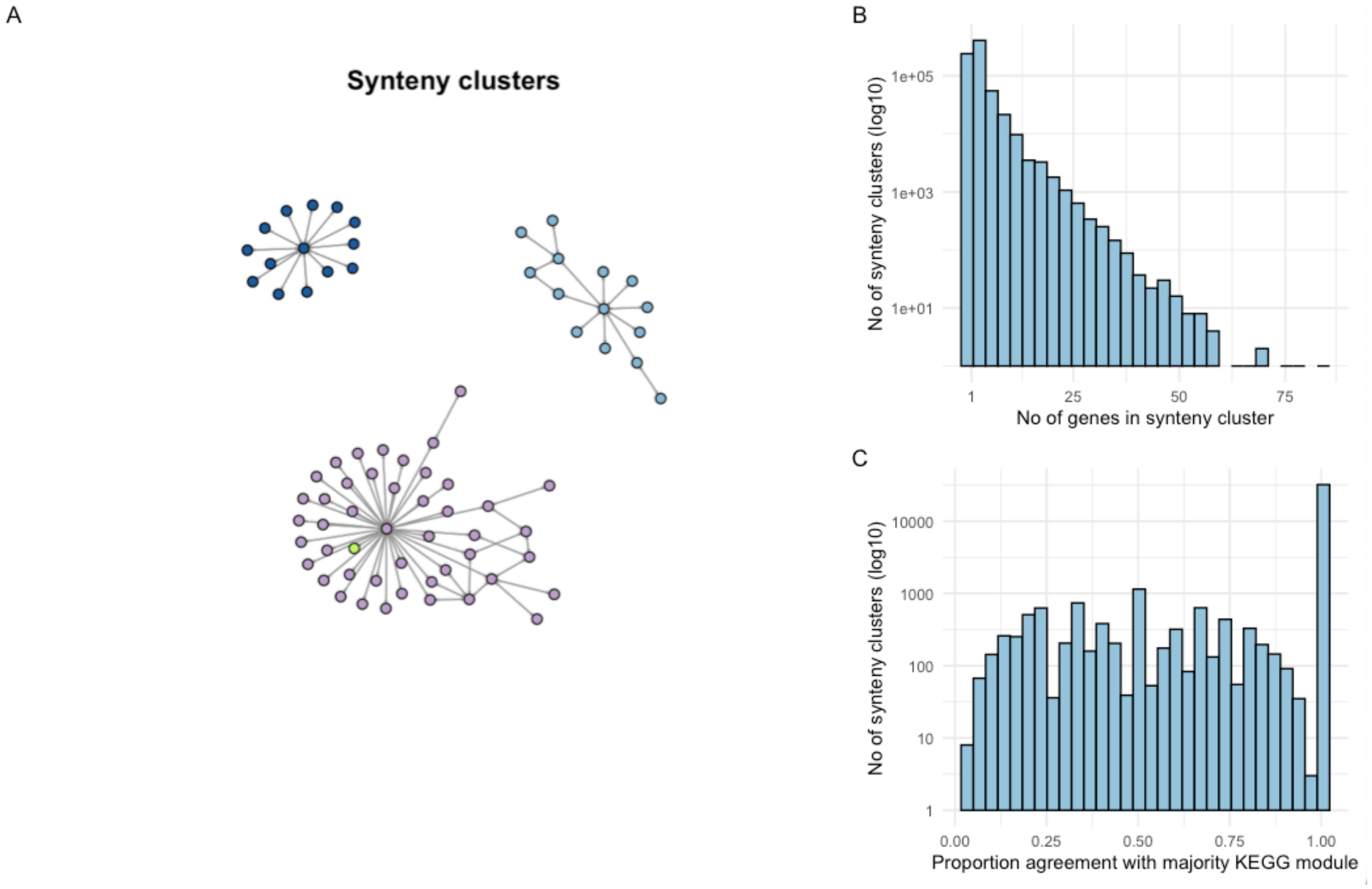

Supplementary Figure 8: *Synteny clusters and functional annotation of* $COPSAC_{2010}$

A) The graph network of 3 synteny clusters are shown. The colours represent KEGG modules (green indicates no KEGG module annotation). B) The distribution of synteny cluster size. C) The proportion of genes in the synteny cluster in agreement with the most common KEGG module in the cluster. Only synteny clusters with 5 or more genes are included. Source data are provided as a Source Data file.

***Supplementary Table 1**: Output generated by MAGinator*

**Directory**	**Content**
abundance	abundance_phyloseq.RData - Phyloseq object for R, with abundance and taxonomic data
clusters	.fa - Fasta files with nucleotide sequence of bins
genes	all_genes.faa - Amino acid sequences of all ORFs<br>all_genes.fna - Nucleotide sequences of all ORFs<br>all_genes_nonredundant.fasta - Nucleotide sequences of gene cluster representatives<br>all_genes_cluster.tsv - Gene clusters<br>matrix/gene_count_matrix.tsv - Read count for each gene cluster for each sample<br>synteny/ - Intermediate files for synteny clustering of gene clusters
gtdbtk	GTDB-tk taxonomic annotation for each MAG cluster
logs	Log files
mapped_ reads	bams/ - Bam files for mapping reads to gene clusters
phylo	alignments/ - Alignments for each signature gene<br>cluster_alignments/ - Concatenated alignments for each MAG cluster<br>pileup/ - SNV information for each MAG cluster and each sample<br>trees/ - Phylogenetic trees for each MAG cluster<br>stats.tab - Mapping information such as non-N fraction, number of signature genes and marker genes, read depth, and number of bases not reaching allele frequency cutoff<br>stats_genes.tab - Same as above but the information is split per gene
signature_g enes	R data files with signature gene optimization<br>read-count_detected-genes.pdf - Figure for each MAG cluster displaying number of identified SG's in each sample along with the number of reads mapped.
tabs	gene_cluster_bins.tab - Table listing which bins each gene cluster was found in<br>gene_cluster_tax_scope.tab - Table listing the taxonomic scope of each gene cluster<br>metagenomicspecies.tab - Table listing which, if any, clusters where merged in MAG cluster and the taxonomy of those<br>signature_genes_cluster.tsv - Table with the signature genes for each MAG cluster<br>synteny_clusters.tab - Table listing the synteny cluster association for the gene clusters. Gene clusters from the same synteny cluster are genomically adjacent.<br>tax_matrix.tsv - Table with taxonomy information for MAG cluster

Supplementary Table 2: Parameters and default values of MAGinator

Argument	Description	Default Value
binsize	Minimum bin size for inclusion.	200000
mgs_collections	If set, bin clusters will be aggregated to metagenomic species.	-
annotation_prevalence	Minimum prevalence of taxonomic assignment in a cluster of bins to call consensus.	0.75
clustering_coverage	Alignment coverage for clustering of genes with MMseqs2.	0.8
clustering_min_seq_id	Sequence identity threshold for clustering of genes with MMseqs2.	0.95
clustering_type	Sequence type for gene clustering with MMseqs2. Nucleotide- or protein-level.	protein
min_gtdb_markers	Minimum GTDBtk marker genes shared between MGS and outgroup for rooting trees.	10
marker_gene_cluster_prevalence	Minimum prevalence of marker genes to be selected for rooting of MGS trees.	0.5
min_mapped_signature_genes	Minimum number of signature genes with reads mapped for the sample to be included in the refinement.	3
min_samples	Minimum number of samples containing the MAG cluster for the MAG cluster to identify SG.	3
min_af	Minimum allele frequency for calling a base when creating phylogenies.	0.8
min_depth	Minimum read depth for calling a base when creating phylogenies.	2
min_nonN	Minimum fraction of non-N characters of a sample to be included in a phylogeny.	0.5
min_marker_genes	Minimum marker genes to be detected for inclusion of a sample in a phylogeny.	2
min_signature_genes	Minimum signature genes to be detected for inclusion of a sample in a phylogeny.	50
af_cutoff	Cutoff for average median allele frequency of SG alignment for determining mixed/pure population of a MAG in a sample	0
phylo	Software for phylogeny inference. Either fast (fasttree) or slow and more accurate (iqtree).	fasttree
tax_scope_threshold	Threshold for assigning the taxonomic scope of a gene cluster.	0.9

synteny_adj_cutoff	Minimum number of times gene clusters should be adjacent to be included in synteny graph.	1
synteny_mcl_inflation	Inflation parameter for mcl clustering of synteny graph. Usually between 1.2 and 5. Higher values produce smaller clusters.	5

Supplementary Table 3: *OPAL benchmark. Average completeness (%) across taxonomic ranks.*

The mean of the tools are indicated with bold.

	superkingdom	phylum	class	order	family	genus	species	strain
**Mean across tools**	**100**	**90.1**	**75.1**	**79.0**	**83.3**	**82.7**	**45.6**	**0.7**
DUDes	100	67	53.6	61.3	55.1	60.2	32.4	0
DUDes cami1	100	98.8	81.6	68.8	95.7	91.8	63.4	0
LSHVec gsa	100	50	40	50	33.3	33.3	15	0
MAGinator	100	99.5	99	83	88	99.8	89.6	7.4
MetaPhlAn	100	96	76.8	80.7	84.3	89.2	67.1	0
MetaPhlAn_cami1	100	94.8	75.8	79.8	81.8	71.6	29.4	0
MetaPhyler	100	97	80	83.3	94.2	92.2	0	0
mOTUs 2.0.1_1	100	94.2	60	79.5	90.8	85.7	0	0
mOTUs 2.5.1_6	100	97	81.6	84.7	85.9	93.6	84	0
mOTUs cami1	100	97	78	81.7	96	92.9	67.4	0
TIPP cami1	100	100	100	100	100	99.5	53.8	0

Supplementary Table 4*: OPAL benchmark. Average purity (%) across taxonomic ranks.*

The mean of the tools are indicated with bold.

	superkingdom	phylum	class	order	family	genus	species	strain
**Mean across tools**	**95.2**	**92.4**	**85.9**	**79.9**	**80.0**	**73.6**	**36.5**	**8.8**
DUDes	100	100	100	100	98.1	95.3	84.9	0
DUDes cami1	100	100	80.6	61.1	84.3	57.8	34.9	0
LSHVec gsa	100	100	100	100	100	100	37.5	0
MAGinator	100	100	100	84	80	80	90.1	96.7
MetaPhlAn	100	100	100	100	88.5	92.8	49.5	0
MetaPhlAn cami1	97	100	100	100	99.3	91.1	63.8	0
MetaPhyler	100	100	97.4	72.7	78.9	79.2	0	0
mOTUs 2.0.1_1	100	100	100	100	100	90.9	0	0
mOTUs 2.5.1_6	100	100	60.6	51.5	43	37.3	17.8	0
mOTUs cami1	100	100	93.2	87.4	91.7	69.1	21	0
TIPP cami1	50	16.9	12.6	6.1	5.43	4.21	2.43	0

Supplementary Table 5: *Allele Frequency results for different metagenomics datasets*

	Number of samples	MAG clusters	MAG clusters with mixed alleles	Total number of samples/leaf tips	Total number of samples with mixed alleles	Percent of samples in MAG clusters with mixed alleles
COPSAC 2010	662	716	329 (46%)	20,765	4,154 (20%)	38%
Tara Oceans	148	791	540 (68%)	10,805	3,523 (33%)	37%
Honey Bee	54	195	168 (86%)	6,035	4,223 (70%)	71%
